# Comparative Analysis of Oral Bacterial Profiles in Parkinson’s Disease According to Periodontal Status: A Clinical Case Series

**DOI:** 10.3390/healthcare14030362

**Published:** 2026-01-30

**Authors:** Dragoș Nicolae Ciongaru, Silviu Mirel Piţuru, Stana Păunică, Marina Cristina Giurgiu, Ioana Bujdei-Tebeică, Anca-Silvia Dumitriu

**Affiliations:** 1 Department of Periodontology, Faculty of Dental Medicine, Carol Davila University of Medicine and Pharmacy, 030167 Bucharest, Romania; nicolae-dragos.ciongaru@drd.umfcd.ro (D.N.C.); stana.paunica@umfcd.ro (S.P.); anca.dumitriu@umfcd.ro (A.-S.D.); 2 Department of Professional Organization and Medical Legislation-Malpractice, Faculty of Dental Medicine, Carol Davila University of Medicine and Pharmacy, 030167 Bucharest, Romania; silviu.pituru@umfcd.ro; 3 Department of Diabetes, Nutrition and Metabolic Diseases, Faculty of Medicine, Carol Davila University of Medicine and Pharmacy, 030167 Bucharest, Romania; ioana.paunica@drd.umfcd.ro

**Keywords:** Parkinson’s disease, dysbiosis, periodontal disease, periodontal pathogens

## Abstract

**Introduction**: Parkinson’s disease can influence oral health by impairing motor function and altering salivary composition, potentially affecting the oral microbiome. **Materials and Methods**: The objectives of this study are fourfold: (a) to compare the prevalence of bacterial species associated with periodontal disease in patients with and without Parkinson’s disease (PD), (b) to assess whether the coexistence of periodontal disease in PD patients contributes to an imbalance in the oral microbiome, (c) to evaluate the correlation between periodontal clinical indices (plaque index, tartar index, bleeding index, and probing depth) and the concentrations of specific periodontopathogenic bacterial species, and (d) to explore the potential implications of these evidences for clinical management and preventive strategies in Parkinson’s patients. The main objective of this study is to compare periodontal clinical indices (plaque index, tartar index, bleeding index, and probing depth) and the bacterial profile of patients with periodontal and Parknson’s disease. Two groups were included: 15 patients with periodontal disease (control group) and 16 patients with both periodontal and Parkinson’s disease (study group). Microbial samples were collected from the periodontal pockets at baseline and analyzed using the Polymerase Chain Reaction (PCR) Perio-Ident 12 kit to detect major periodontal pathogens. **Results**: Periodontal indices showed no statistically significant differences between groups, although the study group presented lower mean tartar index (49.31% vs. 67.4%, *p* = 0.069), bleeding on probing (44.31% vs. 56.67%, *p* = 0.137), and plaque index (66% vs. 68.93%, *p* = 0.754). Median bacterial loads were generally higher in control group, with *Tannerella forsythia*, but without statistically significant difference (*p* = 0.072). Significant correlations between plaque index and multiple pathogens occurred only in control gorup, suggesting disrupted plaque–pathogen dynamics (*p* < 0.05). **Conclusions**: The results highlight the potential value of integrating clinical and microbiological assessment when managing periodontal disease in patients with Parkinson’s disease.

## 1. Introduction

Parkinson’s disease (PD) is a progressive neurodegenerative disorder primarily characterized by motor symptoms tremor, rigidity, bradykinesia, and postural instability. In addition to these motor impairments, PD is associated with a wide range of non-motor manifestations such as autonomic dysfunction and cognitive decline [1] (p. 18). An emerging area of research concerns the potential relationship between the disease and oral health, particularly the bacterial profile of the oral cavity [1,2,3] (pp. 2, 7, 19). As PD advances, its impact on oral health can become substantial due to reduced manual dexterity affecting oral hygiene, alterations in salivary composition, and increased susceptibility to infections, including periodontal disease [4] (p. 434).

Periodontal disease is a chronic inflammatory condition affecting the supporting structures of the teeth, including the gingiva, periodontal ligament, and alveolar bone. It is primarily caused by an imbalance in the oral microbiome that favors the proliferation of pathogenic bacteria, ultimately contributing to progressive tissue destruction. Among the bacterial species commonly associated with periodontal disease are those classified within Socransky’s periodontal complexes. In particular, the red complex bacteria- comprising *Porphyromonas gingivalis*, *Treponema denticola,* and *Tannerella forsythia*—is considered highly pathogenic and virulent and is strongly associated with severe periodontal breakdown [5,6,7] (pp. 7, 9, 10). *Porphyromonas gingivalis*, a major etiological agent of periodontitis, produces highly immunogenic lipopolysaccharide (LPS) endotoxins capable of initiating systemic inflammatory responses [8] (p. 12). Growing evidence suggests that LPS originating in the oral cavity can disseminate systemically and reach the central nervous system, where it may trigger microglial activation and sustain neuroinflammatory cascades [9] (p. 2).

Chronic neuroinflammation is a well-established pathogenic contributor to neurodegenerative disorders, including Parkinson’s disease. Experimental studies suggest that LPS promotes α-synuclein aggregation, a defining neuropathological feature of Parkinson’s disease. Moreover, LPS-mediated oxidative stress and mitochondrial dysfunction further increase the vulnerability of dopaminergic neurons [10] (p. 2). In Parkinson’s disease, LPS from *P. gingivalis* may amplify neuronal damage by enhancing apoptosis, partly through dysregulation of the protein Kinase B (AKT) and mechanistic target of rapamycin (mTOR) signaling pathway. Impaired AKT–mTOR activity reduces cell-survival signaling and increases susceptibility of dopaminergic neurons to degeneration, potentially accelerating disease progression [11,12] (pp. 10, 12).

Recent studies further support the role of the oral microbiome in Parkinson’s disease. A large metagenomic analysis showed that PD patients present distinct alterations in both the oral and gut microbiome. Another study demonstrated that microbial virulence factors and functional dysbiosis may be linked to cognitive decline in PD, suggesting a possible microbiome-mediated influence on neurodegenerative mechanisms. Additionally, sex-specific differences in the salivary microbiome were identified, indicating that biological sex may modify microbial composition and potentially influence symptom variability in Parkinson’s disease [2,13,14] (pp. 2, 5, 7).

This findings support a possible link between periodontal infection and Parkinsonian neurodegeneration.

Although a causal relationship has not been definitively established, the association underscores the relevance of oral–systemic interactions in the pathophysiology of neurodegenerative disease. The relationship between PD and periodontal disease is multifactorial. PD-related motor impairments can reduce effective oral hygiene practices, thereby promoting bacterial accumulation and increasing the risk of periodontal inflammation [15,16] (pp. 2, 5).

Given these factors, understanding the bacterial profile of PD patients with and without periodontal disease is essential for developing targeted interventions to improve oral and overall health outcomes. Previous research have examined the effects of neurodegenerative diseases on oral health, with findings suggesting that PD patients exhibit a higher prevalence of periodontal disease compared with age-matched controls without PD [15,16,17] (pp. 2, 9). However, there remains a gap in the literature regarding the specific bacterial composition of the oral microbiome in PD patients and how it differs based on periodontal disease status. Although several cross-sectional and sequencing-based studies have described global alterations of the oral and gut microbiome in Parkinson’s disease, relatively limited attention has been given to the periodontal pathogens in relation to clinical periodontal parameters. Most microbiome-based investigations rely on broad taxonomic profiling, which may obscure the role of specific high-virulence bacteria directly implicated in periodontal tissue destruction and systemic inflammatory signaling. In this context, the present study addresses a distinct knowledge gap by applying a targeted, pathogen-specific qPCR approach to quantify major periodontopathogenic species and to examine their relationship with periodontal clinical indices in patients with and without Parkinson’s disease. By focusing on clinically relevant periopathogens, this study provides clinically actionable insights into Parkinson’s disease–associated oral dysbiosis.

Several mechanistic links between periodontal pathogens, systemic inflammation, and neurodegenerative processes have been proposed, largely based on experimental and preclinical studies. These include the potential role of lipopolysaccharide-mediated inflammation, immune dysregulation, and microbiota–host interactions within the oral–gut–brain axis. While such mechanisms provide important biological context, they cannot be directly examined within the framework of the present cross-sectional, clinically oriented study. Accordingly, the current investigation is not designed to test pathways but rather to provide a descriptive clinical and microbiological comparison of periodontal status and selected oral bacterial profiles in patients with and without Parkinson’s disease. The mechanistic concepts discussed herein are therefore intended to contextualize the clinical observations and to support hypothesis generation for future longitudinal and experimental research.

The aim of this study is to investigate the relationship between Parkinson’s disease and periodontal status by comparing periodontal clinical indices and oral bacterial. Specifically, we evaluated the prevalence and concentration of periodontopathogenic bacterial species and analyzed their correlations with clinical periodontal parameters, in order to assess potential microbiome imbalances and their implications for clinical management and preventive strategies in Parkinson’s disease.

Overall, this comparative study seeks to advance our understandings of the bacterial profile in PD. The results are expected to highlight the importance of interdisciplinary approaches to patient care, integrating dental and neurological expertise to optimize health outcomes for individuals living with PD.

## 2. Materials and Methods

### 2.1. Study Design and Objectives

This study was designed as a cross-sectional, exploratory clinical case series. Given the limited final sample size, the analyses were intended to provide descriptive and exploratory insights rather than confirmatory or causal inferences. Also, the study was conducted to evaluate the association between periodontal status and Parkinson’s disease. Due to the nature of patient interaction, neurological assessment, and the need to obtain comprehensive medical histories, blinded study was not possible. Periodontal microbial analysis was performed using the PCR Perio-Ident 12 test, a molecular diagnostic method designed to detect bacterial species associated with periodontal disease.

The following periodontal pathogens were analyzed: *Aggregatibacter actinomycetemcomitans*, *Porphyromonas gingivalis*, *Treponema denticola*, *Tannerella forsythia*, *Eikenella corrodens*, *Campylobacter rectus*, *Prevotella intermedia*, *Fusobacterium nucleatum*, *Prevotella nigrescens*, *Capnocytophaga ochracea*, *Capnocytophaga sputigena*, and *Capnocytophaga gingivalis*.

### 2.2. Ethical Considerations

The study protocol was approved by the Scientific Research Ethics Commission of “Carol Davila” University of Medicine and Pharmacy, Bucharest, Romania (protocol no. 24376/23 September 2025), and was conducted in accordance with the principles of the Declaration of Helsinki (1975, as revised). Written informed consent was obtained from all participants. All patients demonstrated sufficient cognitive capacity and motor function to understand the study procedures and to personally sign the consent form.

### 2.3. Study Participants

The study included two groups of patients:

Control Group: Patients with periodontal disease and without Parkinson’s disease.

Study Group: Patients with both Parkinson’s disease and periodontal disease.

The diagnosis of Parkinson’s disease was established by a specialist neurologist. The diagnosis of periodontal disease was established by a specialist periodontist, following the European Federation of Periodontology (EFP) 2018 classification criteria [18] (pp. 4–5).

#### 2.3.1. Inclusion Criteria

- Patients diagnosed with periodontal disease or Parkinson’s disease.
- Cooperative patients of both sexes, aged 18–80 years.
- No systemic comorbidities (diabetes mellitus, cardiovascular disease or chronic hepatic conditions).
- No periodontal treatment within previous year.
- No dental implants or prosthetic restorations.
- No antibiotic treatment within previous year.

#### 2.3.2. Exclusion Criteria

- Failure to meet inclusion criteria.
- Presence of acute periodontal conditions (abscess, pericoronitis).
- Severe motor deficits preventing adequate oral hygiene.
- Ongoing psychiatric treatment antiepileptics, (monoamine oxidase inhibitors) or chronic anti-inflammatory/corticosteroid therapy.
- Antibiotic use within the previous year or antibiotic allergy.
- Pregnancy.
- Presence of chronic systemic diseases (e.g., cardiovascular diseases, diabetes mellitus, neoplasms, autoimmune disorders, cirrhosis, acute infections).

A substantial number of screened patients were excluded based on strict inclusion and exclusion criteria, resulting in a highly selected study population. While this approach increased internal consistency, it may limit representativeness and external validity.

Given the exploratory nature of this observational study, the sample size was limited by the availability of eligible participants meeting strict inclusion and exclusion criteria. While sufficient for descriptive and correlation analyses, the relatively small cohort limits statistical power and precludes strong causal inference. This constraint was considered a priori and is acknowledged as an inherent limitation of the study design.

### 2.4. Data Collection

#### 2.4.1. Demographic Data

Demographic data, including age and sex, were obtained through anamnesis and documented for all participants to ensure proper characterization of the study sample. According to medical records, all patients with Parkinson’s disease had been diagnosed by a specialist neurologist following a comprehensive neurological evaluation confirming symptoms consistent with PD. All patients diagnosed with Parkinson’s disease were undergoing dopamine-based pharmacological therapy, specifically levodopa–carbidopa. Medication status was recorded as clinical background information and was not analyzed as an independent variable in the statistical analyses.

#### 2.4.2. Clinical Periodontal Indices

All clinical examinations were performed by a single experienced periodontist. At baseline, the following clinical periodontal parameters were assessed: bleeding index, plaque index, tartar index, and probing depth [19,20,21] (pp. 10, 12). All clinical measurements were performed by the same calibrated examiner using a North Carolina 15 mm periodontal probe. All clinical periodontal examinations were conducted by a single calibrated periodontist to ensure measurement consistency. As only one examiner was involved, inter-examiner reliability assessment was not applicable.

Bleeding Index (bleeding on probing): evaluates the presence or absence of bleeding following probing of the periodontal pocket. Plaque Index: assesses the visible accumulation of dental plaque on tooth surfaces. Calculus (Tartar) Index: evaluates the extent of mineralized deposits (calculus) present on tooth surfaces. The plaque, tartar, and bleeding indices were calculated as percentage values, based on four dental surfaces per tooth (mesial, distal, buccal, and oral). Each index was determined by dividing the number of affected surfaces by the total number of examined surfaces and multiplying the result by 100. Probing Depth: indicates the depth of the periodontal pocket. Probing depth was measured at six sites per tooth—mesiobuccal, mid-buccal, distobuccal, mesio-oral, mid-oral, and disto-oral. The probing depth is measured from the level of the gingival margin to the most apical point of the periodontal pocket.

For analytical reference, normal clinical values were considered according with EFP guideline as follows: plaque index < 20%, calculus (tartar) index < 10%, bleeding on probing <10% of sites, and physiological probing depth between 1–3 mm. These reference ranges were used to contextualize and interpret the correlation analyses between periodontal indices and microbial load [22] (pp. 15–16).

According to the European Federation of Periodontology (EFP) 2018 classification criteria based on: bleeding on probing greater than 30% and a mean probing depth of 5 mm, all patients were classified as having periodontitis.

#### 2.4.3. Microbiological Analysis

The microbiological assessment was based on a targeted, semi-quantitative PCR panel designed to detect selected periodontal pathogens. This approach does not provide a comprehensive characterization of the overall oral or subgingival microbiome but focuses on clinically relevant periopathogenic species.

##### Collection of the Gingival Crevicular Fluid

Gingival crevicular fluid (GCF) sampling was performed in accordance with the instructions provided by the laboratory and the kit manufacturer. GCF samples were collected from periodontal pockets by a periodontal specialist using a sterile collection kit containing five paper points and a transfer tube. Each sterile paper point was gently inserted into a different periodontal pocket for approximately 10 s to absorb the GCF. After collection, the paper points were placed into the sterile transfer tube and transported to the laboratory for DNA extraction and bacterial load analysis.

##### Bacterial DNA Extraction

The paper points were stored in 1 mL of 1–1.5× phosphate-buffered saline (PBS) at 4–8 °C. This was followed by an additional incubation of approximately 4 h at 37–40 °C with agitation to maximize extraction efficiency. The liquid from each tube was then completely transferred to a new 1.5 mL microcentrifuge tube and centrifuged for 10 min at 14,000 rpm. The resulting pellet, together with approximately 100–150 µL of PBS, was subsequently processed using a genomic DNA extraction kit (Favorgen Biotech Corp, Hilden, Germany) in accordance with the manufacturers’ instructions.

##### Quantification by Real-Time PCR (qRT-PCR)

Bacterial quantification was performed using a Bio-Rad CFX-96 Real-Time PCR system with CFX Maestro 2.0 software (Bio-Rad Laboratories, Hercules, CA, USA). Species-specific reaction mixtures were prepared in 96-well plates, each containing Qiagen RT^2^ SYBR Green ROX qPCR Mastermix (Qiagen, Hilden, Germany), forward and reverse primers, and template DNA, for a final reaction volume of approximately 20 µL. Primers were selected from the literature, validated, and updated according to the latest Ensembl database (2023) [23,24] (pp. 4, 21). The amplification protocol consisted of an initial denaturation at 95 °C for 5 min, followed by 40 cycles of denaturation at 95 °C for 10 s, annealing at 60 °C for 5 s, and extension at 72 °C for 25 s, with fluorescence acquisition. High-resolution melting (HRM) analysis was performed to confirm species-specific amplicons. Relative quantification was conducted using a standard curve generated from *Porphyromonas gingivalis* genomic DNA, calibrated to 6.0 × 10^6^ colony-forming units (CFUs) [24,25] (pp. 21, 49–55).

##### Principle of the Detection

The Perio-Ident 12 genetic test is based on species-specific amplification of genetic material from 12 periodontal bacterial strains using real-time PCR (RT-qPCR), with specificity confirmed by high-resolution melting (HRM) analysis. Amplification was monitored using SYBR Green fluorescence, and results were evaluated based on cycle threshold (Ct) values, defined as the cycle at which fluorescence exceeded background levels (~1000 RFU). Only reactions with Ct < 36 were considered positive to minimize false-positive results.

Post-amplification specificity was verified by HRM analysis, which involved gradual denaturation of PCR amplicons and assessment of fluorescence dissociation curves. Melting temperature (Tm) values were identified from dissociation peaks corresponding to the maximum rate of fluorescence decrease. Each bacterial species was identified based on its characteristic Tm derived from species-specific 16S rRNA amplicons; samples with atypical melting profiles were excluded from analysis.

Bacterial load was estimated using relative quantification based on standard curves generated from serial dilutions of genomic DNA extracted from pure cultures, starting with *Porphyromonas gingivalis* at 6.0 × 10^6^ colony-forming units (CFUs). Comparable standard curves were obtained for other representative species. Samples showing non-specific amplification or Ct values ≥ 36 were classified as negative, while valid positive samples were reported with their corresponding Ct values. Data were exported to Microsoft Excel for numerical and graphical analysis. Bacterial concentrations ≥ 10^3^ CFU for *Aggregatibacter actinomycetemcomitans*, ≥10^4^ CFU for *Porphyromonas gingivalis*, *Treponema denticola*, and *Tannerella forsythia*, ≥10^5^ CFU for *Eikenella corrodens*, *Campylobacter rectus*, *Prevotella intermedia*, *Fusobacterium nucleatum*, *Fusobacterium periodonticum*, and *Prevotella nigrescens*, and ≥10^6^ CFU for *Capnocytophaga* spp. were considered indicative of periodontal pathogenic risk.

##### Statistical Analysis

All data were analyzed using IBM SPSS Statistics version 25 (IBM Corp., Armonk, NY, USA) and illustrated using Microsoft Office Excel and Word 2025 (Microsoft Corp., Redmond, WA, USA). Quantitative variables were assessed for normality using the Shapiro–Wilk test and are presented as means ± standard deviations or medians with interquartile ranges, as appropriate. Qualitative variables are expressed as counts and percentages and were compared between groups using Fisher’s exact test. Quantitative variables with a normal distribution were compared between groups using Student’s *t*-test, with variance homogeneity assessed by Levene’s test, whereas non-normally distributed quantitative variables were analyzed using the Mann–Whitney *U* test. Correlations between non-normally distributed quantitative variables (bacterial concentrations and periodontal indices) were evaluated using Spearman’s rank correlation coefficients. Given the small sample size and the semi-quantitative nature of the bacterial data, all correlation analyses were performed for exploratory purposes and should be interpreted accordingly.

No a priori sample size or statistical power calculation was performed, as the study was designed as an exploratory observational analysis based on the available eligible patient population. This aspect is acknowledged as a methodological limitation.

## 3. Results

A total of 120 patients presented to the Clinical Department of Periodontology. Based on the selection criteria, 50 potential participants were identified. Of these, 31 met all inclusion and exclusion criteria and were enrolled: 16 with periodontal disease and Parkinson’s disease, and 15 with periodontal disease alone.

Patients were excluded due to acute disease requiring antibiotic or anti-inflammatory therapy—such as acute periodontal abscess (4 cases), pericoronitis (2 cases), acute sinusitis (1 case), bacterial respiratory infections (4 cases), or acute odontogenic infections (2 cases) (n = 13)—as well as lack of cooperation (6 cases).

**Figure 1:**
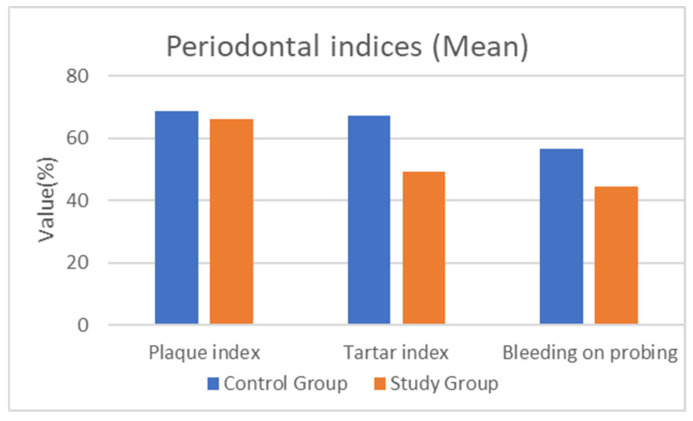
Comparison of periodontal inflammation indices between the two groups: tartar (67.4% vs. 49.31%), plaque levels (68.93% vs. 66%), and bleeding index (56.67% vs. 44.31%).

A total of 31 participants were included in the study, 15 (48.4%) with periodontal disease and 16 (51.6%) with both periodontal disease and Parkinson’s disease (PD). Although the periodontal inflammation indices did not show statistically significant differences between groups, patients in the control group present higher clinical values across most parameters (Figure 1).

The tartar index showed lower mean values in the study group (49.31 ± 28.11%) compared to the control group (67.4 ± 25.03%, *p* = 0.069). Similarly, the bleeding index and plaque index were reduced in the study group (44.31 ± 22.13% and 66 ± 26.74%, respectively) versus the control group (56.67 ± 22.83% and 68.93 ± 24.66%), reflecting altered gingival response and oral self-care in Parkinson’s patients. Lower mean bleeding index values were observed in the study group; however, these differences did not reach statistical significance (Table 1).

Regarding microbiological parameters, the median concentrations of key periodontal pathogens were higher in the control group compared with the study group. Notably, *Tannerella forsythia* exhibited a difference (*p* = 0.072), with reduced median concentrations in study group patients (35,950 CFU/mL) compared to those with control group (200,000 CFU/mL). Although no statistically significant between-group differences were observed for the analyzed bacterial species, the overall microbial profile of patients in the study group showed descriptive variability in both bacterial diversity and bacterial load, which may be related to disease-associated changes in the oral microenvironment and motor limitations affecting oral hygiene.

The pathogen risk analysis revealed that most patients from both groups presented a high-risk bacterial profile, but the proportion was lower in the study group (68.7%) compared to the control group (80%).

In the control group, the plaque index is correlated with the concentrations of *Eikenella corrodens*, *Fusobacterium nucleatum*, *Prevotella nigrescens*, *Capnocytophaga ochracea*, and *Capnocytophaga gingivalis* (all *p* < 0.05) (Table 2). In contrast, in the study group, these relationships were not observed, indicating a disrupted connection between plaque formation and bacterial proliferation.

Data from Table 2 show the correlations between periodontal inflammation analyzed indices and pathogen concentrations in control group patients.

Significant correlations were observed for the plaque index and bacterial concentration of *Eikenella corrodens* (*p* = 0.008, R = 0.659), *Fusobacterium nucleatum* (*p* = 0.007, R = 0.667), *Prevotella nigrescens* (*p* = 0.021, R = 0.589), *Capnocytophaga ochracea* (*p* = 0.012, R = 0.628) and *Capnocytophaga gingivalis* (*p* = 0.007, R = 0.667), being significant high-grade positive correlations. This aspect show that patients with higher plaque index were significantly more associated with also higher bacterial concentrations of those species and viceversa. Additionally, positive correlations were observed between probing depth and the bacterial concentrations of *Campylobacter rectus* (*p* = 0.055, R = 0.505) and *Capnocytophaga ochracea* (*p* = 0.078, R = 0.467); however, these associations did not reach statistical significance.

Data from Table 3 show the correlations between peridontal indices and pathogen concentrations in study group patients. None of the analyzed correlations were statistically significant, only a difference was observed for the correlations between bleeding index and bacterial concentration of *Campylobacter gingivalis* (*p* = 0.098, R = −0.428) towards observing higher values of bacterial concentrations in cases with lower bleeding index and viceversa.

## 4. Discussion

In relation to recent systematic reviews on periodontitis in Parkinson’s disease, our study provides additional value by offering quantitative, pathogen-specific microbiological data—an element identified as lacking in the current literature While reviews emphasize the higher periodontal risk in PD, they also note the scarcity of studies examining microbial patterns. Our exploratory evidence of associations between Parkinson’s disease and oral microbial patterns confirm this knowledge gap and show that PD patients exhibit a disrupted relationship between plaque accumulation and pathogen load, supporting the hypothesis of PD-associated oral dysbiosis. Thus, the study strengthens existing evidence by suggesting that PD influences not only clinical periodontal outcomes but also the ecological behavior of periodontal pathogens.

The inclusion and exclusion criteria in the current study were applied identically for both the control group (periodontal disease without Parkinson’s disease) and the study group (periodontal disease with Parkinson’s disease), with the only distinguishing factor being the confirmed neurological diagnosis of Parkinson’s disease by a specialist neurologist in the study group. Periodontal disease was diagnosed according to the 2018 European Federation of Periodontology (EFP) 2018 Classification System, based on the presence of periodontal pocket depth ≥ 4 mm, bleeding on probing > 30%. In this study, the term “systemic comorbidities” refers to chronic medical conditions known to influence periodontal inflammation—such as diabetes mellitus, cardiovascular disease or chronic hepatic conditions—and corresponds directly to the exclusion criteria established for both groups. Severe motor deficits were defined as functional impairments consistent with postural instability markedly interfered with the patient’s ability to maintain oral hygiene or to cooperate reliably with periodontal examination. Although this criterion was applied to ensure reliable periodontal examination and adequate patient cooperation, it may limit the generalizability of the results. Although no statistically significant differences were observed in most clinical periodontal indices, the microbiological data obtained through the Perio-Ident 12 PCR analysis indicate that Parkinson’s disease may modify the bacterial flora of the oral cavity. Thus, the study reinforces that Parkinson’s disease not only affects motor function but also modulates oral microbial behavior, ultimately placing patients at greater risk for more severe periodontal deterioration than their non-PD counterparts.

An important consideration when interpreting these exploratory evidence is their relevance to the use of adjunctive antibiotic therapy in periodontal management, particularly in cases characterized by elevated bacterial loads and a microbiological profile dominated by highly virulent periopathogens such as *Porphyromonas gingivalis*, *Treponema denticola*, and *Tannerella forsythia*. In the present study, both groups exhibited a high-risk microbial profile; however, patients with Parkinson’s disease showed a greater bacterial diversity and a disrupted relationship between clinical plaque accumulation and bacterial concentration. These findings support evaluating whether selected patients—particularly those with persistent inflammation and elevated pathogen levels—may benefit from carefully targeted adjunctive antibiotic therapy. According to the European Federation of Periodontology guidelines, systemic antibiotics should not be routinely prescribed for the management of periodontitis. Their use is reserved for specific indications, including advanced stages of disease (stage III–IV periodontitis), presence of highly aggressive pathogens, inadequate response to non-surgical periodontal therapy, or systemic conditions that impair host immunity [22] (pp. 9, 29).

In patients with both periodontal disease and Parkinson’s disease, the decision to prescribe antibiotics becomes particularly relevant. The altered microbiological patterns identified in the PD group may necessitate individualized therapeutic strategies when conventional therapy proves insufficient, especially in the presence of red-complex pathogens known to exacerbate periodontal inflammation and potentially contribute to systemic inflammatory pathways associated with neurodegeneration.

### 4.1. Study Limitations

The limitations identified in the present study should be interpreted in the context of existing literature. Similar investigations examining periodontal status and microbiological profiles in Parkinson’s disease are frequently constrained by small sample sizes, reflecting the clinical and logistical challenges of recruiting well-characterized neurological cohorts. Despite these limitations, converging evidence consistently indicates that patients with Parkinson’s disease exhibit an increased susceptibility to periodontal disease and altered oral microbial ecosystems.

Several limitations should be acknowledged when interpreting the study results and observation. The relatively small number of participants (31 in total) limits the statistical power and may obscure subtle differences between groups, particularly regarding low-prevalence bacterial species. A larger cohort would be required to confirm the observed trends. The absence of an a priori power calculation further limits the inferential strength of the present findings. Consequently, the results should be interpreted as exploratory and hypothesis-generating rather than confirmatory. Because the study analyzes patients at a single time point, it cannot determine causality between PD and microbiome alterations. Longitudinal observations would be needed to evaluate changes over time and to assess how PD progression affects periodontal biology.

The PCR Perio-Ident 12 test (Hain Lifescience GmbH, Nehren, Germany) is a commercially available molecular diagnostic assay based on real-time polymerase chain reaction (qPCR) technology, designed for the qualitative and semi-quantitative detection of 12 major periodontal pathogens. The test targets species-specific regions of bacterial 16S rRNA genes, allowing sensitive identification of periodontopathogenic microorganisms commonly associated with periodontal disease. The assay is classified as a targeted, pathogen-specific qPCR-based diagnostic test and is widely used in periodontal research and clinical diagnostics. Detection is performed using SYBR Green fluorescence chemistry, with species identification confirmed by high-resolution melting (HRM) curve analysis. Bacterial load estimation is based on cycle threshold (Ct) values and comparison with standardized reference curves, enabling relative quantification of bacterial concentrations. The Perio-Ident 12 test was selected for this study due to its validated sensitivity, reproducibility, and clinical relevance in identifying high-risk periodontal pathogens, particularly red-complex bacteria.

The test, although widely used for identifying major periodontal pathogens, presents several methodological limitations that should be acknowledged. Its diagnostic is restricted to twelve predefined bacterial species, which means that important microorganisms involved in dysbiosis and newly emerging periopathogens are not detected. This limited spectrum may lead to an incomplete representation of the microbial community compared with broader sequencing-based methods. Additionally, the test relies on SYBR Green qPCR, which is susceptible to non-specific amplification and requires careful interpretation of melting curves to avoid false positives. Quantification based on colony-forming unit (CFU) equivalents is also influenced by variable DNA extraction efficiency and differences in bacterial genome copy numbers, reducing absolute accuracy. An additional methodological limitation of the Paro Ident 12 system concerns the inability to perform antibiotic susceptibility testing. Because the method is based exclusively on qPCR detection of bacterial DNA and does not involve bacterial culture, it is not possible to generate antibiograms or evaluate phenotypic antimicrobial resistance profiles. As the kit relies on genetic amplification rather than viable bacterial growth, no isolates are available for culture-based sensitivity analyses. Consequently, the clinician cannot directly determine whether specific pathogens present in the periodontal pockets are susceptible or resistant to particular antibiotic classes. Despite these limitations, several studies have confirmed the clinical utility of targeted qPCR kits in periodontal diagnostics. Masunaga et al. demonstrated that qPCR-based detection offers higher sensitivity than culture methods in identifying red-complex bacteria, while Kirakodu et al. showed that optimized qPCR provides reliable quantification of periodontal pathogens within complex biofilms [23,24,25] (pp. 21, 49–55). Similarly, Feres and Socransky’s validated the relevance of detecting specific high-risk species—such as *Porphyromonas gingivalis*, *Tannerella forsythia*, and *Treponema denticola*—due to their strong association with disease progression [7] (p. 35). These findings support the continued use of Perio Ident 12 as a practical, clinically meaningful tool, particularly when interpreted alongside clinical indices and other microbiological parameters.

All patients with Parkinson’s disease in this study were undergoing dopamine-based therapy, primarily levodopa–carbidopa. These medications may affect salivary flow, mucosal hydration, swallowing frequency, and autonomic regulation [26,27,28] (pp. 15, 40–41). Such changes could indirectly alter oral microbial colonization, plaque accumulation patterns, or gingival inflammatory responses. All patients with Parkinson’s disease included in this research were undergoing dopaminergic therapy with levodopa–carbidopa. Dosages were individualized according to disease severity. Although levodopa–carbidopa represents the cornerstone of Parkinson’s disease management, its influence on salivary secretion and oral homeostasis remains insufficiently understood. Although all Parkinson’s disease patients in this study were undergoing dopaminergic therapy, current evidence does not support a direct and consistent effect of levodopa-based treatment on oral bacterial composition. Therefore, the observed microbiological patterns should be interpreted as multifactorial and not attributable to pharmacological therapy alone. The available studies are limited in number, heterogeneous in methodology, and often report conflicting findings, suggesting that any observed salivary alterations may result from a multifactorial interactions rather than from the medication itself. Given these uncertainties, it is difficult to determine whether the microbiological and periodontal differences observed in this study stem from Parkinson’s disease pathology, the pharmacological regimen, or their combined systemic effects.

Patients with systemic diseases (e.g., diabetes, cardiovascular disorders, autoimmune diseases) were excluded to prevent confounding effects, as these conditions independently modify the oral microbiome and periodontal inflammatory responses. Including them would have made it impossible to attribute microbiological findings specifically to Parkinson’s disease. Similarly, patients who had taken antibiotics within the previous year were excluded because antibiotic exposure can markedly alter oral microbial composition, suppress pathogenic species, and produce long-lasting microbiome shifts. Including such participants would have compromised the accuracy of the bacterial load comparisons and threatened the validity of the microbiological data. Finally, patients receiving chronic anti-inflammatory corticosteroid therapy or psychiatric medication were excluded due to the known immunomodulatory effects of these drugs, which could independently affect periodontal disease severity and bacterial colonization patterns. Patients with advanced motor dysfunction represent a clinically relevant subgroup of Parkinson’s disease and are likely to experience greater difficulties in maintaining oral hygiene, potentially leading to more severe periodontal deterioration and distinct microbial profiles. Therefore, the present results primarily reflect Parkinson’s patients with mild to moderate motor impairment and should be interpreted within this context.

### 4.2. Future Research Directions

The present exploratory evidence of associations between Parkinson’s disease and oral microbial profile underscore the need for future research that more comprehensively integrates microbiological diagnostics into periodontal management protocols, particularly in patients with concomitant Parkinson’s disease. Longitudinal studies with larger cohorts are essential to clarify the temporal dynamics between Parkinson’s disease progression, shifts in oral microbiome composition, pathogen load, and changes in periodontal status. Such studies would help determine whether microbiological alterations appear early in neurodegenerative progression or arise as secondary effects of impaired oral hygiene, altered salivary function, or dopaminergic therapy. Importantly, future research should also differentiate the influence of Parkinson’s pathology itself from the effects of specific dopaminergic regimens. Stratifying patients according to drug type, dosage, treatment duration, timing of administration, and polypharmacy may reveal whether particular pharmacological profiles predispose individuals to distinct microbial patterns or increased periodontal risk.

Another priority for future investigation is the stratification of patients based on periodontitis stage and grade, following the latest EFP classification system. Comparative studies should therefore evaluate treatment outcomes (with and without adjunctive systemic antibiotics) within each stage/grade category, examining how bacterial load reduction, clinical improvement, and microbiome stabilization differ across disease severities.

Future research should also investigate the use of serial microbiological testing during periodontal and antimicrobial treatment, rather than limiting analysis to baseline samples. Repeated microbiological evaluations would provide valuable information regarding the effectiveness of mechanical therapy alone versus combined mechanical–antibiotic therapy. These assessments would allow clinicians to observe real-time changes in microbial composition, detect persistent pathogens, and identify treatment-resistant species. Moreover, such an approach would help determine which antibiotic classes demonstrate the greatest effectiveness against individual or grouped periopathogens (e.g., red-complex bacteria), contributing to evidence-based antibiotic selection tailored to microbiological results.

From a clinical perspective, the study raises questions regarding therapeutic decision-making; however, the present data do not allow conclusions regarding treatment efficacy. Also, the observed dissociation between plaque accumulation and pathogen load in Parkinson’s disease patients raises questions regarding the antibiotic ussage. However, the present data do not allow conclusions regarding therapeutic efficacy or antibiotic indication. Any discussion of adjunctive antibiotic therapy should therefore be interpreted cautiously and within the framework of existing European Federation of Periodontology guidelines. Establishing pathogen-specific antibiotic response profiles may also reduce unnecessary antibiotic usage, aligning with EFP recommendations advocating for judicious, microbiologically justified prescription of systemic antimicrobials. In addition, randomized controlled trials comparing conventional periodontal therapy, adjunctive systemic antibiotics, locally delivered antimicrobials could clarify which interventions are most effective for patients with Parkinson’s disease. These trials should measure not only clinical periodontal outcomes but also systemic inflammatory responses and potential effects on neurological biomarkers. Expanding research to include salivary biomarkers, such as inflammatory mediators, bacterial metabolites may further help distinguish PD-related microbial dysbiosis from patterns typical of periodontal disease alone.

Together, these future research directions will facilitate the development of refined diagnostic frameworks, personalized treatment algorithms, and evidence-based guidelines that integrate clinical, microbiological, and systemic considerations—ultimately improving periodontal and overall health outcomes for patients with Parkinson’s disease.

## 5. Conclusions

This study explore an association between Parkinson’s disease and alterations in the ecological behavior of the oral microbiome, particularly reflected by disrupted relationships between plaque accumulation and periodontal pathogen profile. Although most between-group comparisons did not reach statistical significance, the observed patterns indicate that Parkinson’s disease may be accompanied by qualitative changes in plaque–pathogen dynamics rather than differences in absolute bacterial burden.

Given the limited sample size and cross-sectional design, the present exploratory study should be interpreted as hypothesis-generating rather than confirmatory. Nevertheless, the consistent loss of correlations between clinical periodontal indices and bacterial concentrations in Parkinson’s disease patients highlights the potential limitations of relying solely on traditional clinical parameters for periodontal assessment in this population.

From a clinical perspective, these observations support the need for individualized periodontal management strategies in patients with Parkinson’s disease, integrating clinical examination with microbiological evaluation when appropriate. Further longitudinal and larger-scale studies are required to clarify the clinical significance of these associations and to determine their implications for preventive and therapeutic approaches.

## Figures and Tables

**Table 1:** Comparison of periodontal inflammation indices between the control group and the Parkinson’s disease group. No statistically significant differences were detected between groups for any of the analyzed bacterial species (*p* > 0.05).

Parameter	Control Group	Study Group	*p*
N, %	15 (48.4%)	16 (51.6%)	-
Demographic characteristics			
Age (Median (IQR))	66 (54–69)	59 (51.5–65.5)	0.188 *
Gender (Female) (Nr., %)	10 (66.7%)	10 (62.5%)	1.000 **
Periodontal inflammation indices			
Tartar index (Mean ± SD)	67.4 ± 25.03	49.31 ± 28.11	0.069 ***
Plaque index (Mean ± SD)	68.93 ± 24.66	66 ± 26.74	0.754 ***
Probing depth (Median (IQR))	5 (4–6)	5 (4.25–6)	0.740 *
Bleeding index (Mean ± SD)	56.67 ± 22.83	44.31 ± 22.13	0.137 ***
Periodontal pathogens concentrations (Median (IQR))			
*Aggregatibacter* *Actinomycetemcomitans*	0 (0–0)	0 (0–0)	0.770 *
*Porphyromonas gingivalis*	10,300 (9.8–7,170,000)	14,745 (0–302,000)	0.423 *
*Treponema denticola*	7.02 (0–1570)	470 (0–1785)	0.711 *
*Tannerella forsythia*	200,000 (3400–647,000)	35,950 (1472–155,000)	0.072 *
*Eikenella corrodens*	11,400 (39.9–45,400)	359.5 (40.5–12,872.5)	0.188 *
*Campylobacter rectus*	4650 (41.1–53,800)	2320 (266.75–10,425)	0.318 *
*Prevotella intermedia*	192 (15–1930)	64.8 (0–596.75)	0.425 *
*Fusobacterium nucleatum*	28.3 (0–81.3)	12.7 (1.96–52.03)	0.626 *
*Prevotella nigrescens*	5850 (1320–37,100)	1775 (230.75–41,150)	0.654 *
*Capnocytophaga ochracea*	2510 (285–168,000)	565.5 (135.75–6880)	0.140 *
*Capnocytophaga sputigena*	13,800 (29.2–116,000)	5870 (651.75–93,850)	0.830 *
*Capnocytophaga gingivalis*	23,400 (1800–76,300)	7620 (1562.5–14,375)	0.151 *
Pathogen risk (Nr., %)			
Low	3 (20%)	5 (31.3%)	0.685 **
High	12 (80%)	11 (68.7%)	

* Mann-Whitney *U* Test, ** Fisher’s Exact Test, *** Student *t*-Test.

**Table 2:** Correlations between analyzed indexes and pathogen concentrations in patients with Periodontal disease. Statistically significant positive correlations were observed for specific bacterial species (Spearman’s correlation, *p* < 0.05).

Index/Pathogen *	Tartar Index	Plaque Index	Bleeding Index	Probing Depth
*Aggregatibacter* *Actinomycetemcomitans*	*p* = 0.826 R = −0.062	*p* = 0.373 R = 0.248	*p* = 0.437 R = 0.217	*p* = 1.000 R = 0.000
*Porphyromonas gingivalis*	*p* = 0.116 R = −0.423	*p* = 0.410 R = 0.230	*p* = 0.750 R = 0.090	*p* = 0.276 R = 0.301
*Treponema denticola*	*p* = 0.809 R = −0.068	*p* = 0.457 R = 0.208	*p* = 0.125 R = 0.414	*p* = 0.948 R = 0.018
*Tannerella forsythia*	*p* = 0.341 R = −0.265	*p* = 0.330 R = 0.270	*p* = 0.624 R = −0.138	*p* = 0.320 R = 0.276
*Eikenella corrodens*	*p* = 0.805 R = 0.070	*p* = 0.008 R = 0.659	*p* = 0.239 R = 0.324	*p* = 0.135 R = 0.404
*Campylobacter rectus*	*p* = 0.431 R = −0.220	*p* = 0.133 R = 0.406	*p* = 1.000 R = 0.000	*p* = 0.055 R = 0.505
*Prevotella intermedia*	*p* = 0.334 R = −0.268	*p* = 0.228 R = 0.331	*p* = 0.450 R = 0.211	*p* = 0.528 R = 0.177
*Fusobacterium nucleatum*	*p* = 0.862 R = 0.049	*p* = 0.007 R = 0.667	*p* = 0.392 R = 0.239	*p* = 0.148 R = 0.393
*Prevotella nigrescens*	*p* = 0.638 R = 0.132	*p* = 0.021 R = 0.589	*p* = 0.611 R = 0.143	*p* = 0.106 R = 0.434
*Capnocytophaga ochracea*	*p* = 0.390 R = 0.239	*p* = 0.012 R = 0.628	*p* = 0.357 R = 0.256	*p* = 0.078 R = 0.467
*Capnocytophaga sputigena*	*p* = 0.134 R = −0.406	*p* = 0.273 R = 0.303	*p* = 0.894 R = 0.038	*p* = 0.196 R = 0.354
*Capnocytophaga gingivalis*	*p* = 0.776 R = 0.080	*p* = 0.007 R = 0.667	*p* = 0.223 R = 0.335	*p* = 0.113 R = 0.426

* Spearman’s Correlation Coefficient.

**Table 3:** Correlations between analyzed indexes and pathogen concentrations in patients with Periodontal disease and Parkinson’s disease.

Index/Pathogen *	Tartar Index	Plaque Index	Bleeding Index	Probing Depth
*Aggregatibacter* Actinomycetemcomitans **	-	-	-	-
*Porphyromonas gingivalis*	*p* = 0.320 R = −0.266	*p* = 0.869 R = 0.045	*p* = 0.711 R = 0.100	*p* = 0.541 R = 0.165
*Treponema denticola*	*p* = 0.110 R = −0.415	*p* = 0.869 R = 0.045	*p* = 0.538 R = 0.166	*p* = 0.437 R = 0.209
*Tannerella forsythia*	*p* = 0.249 R = −0.306	*p* = 0.854 R = −0.050	*p* = 0.974 R = 0.009	*p* = 0.977 R = 0.008
*Eikenella corrodens*	*p* = 0.991 R = 0.003	*p* = 0.354 R = 0.248	*p* = 0.771 R = 0.079	*p* = 0.494 R = 0.185
*Campylobacter rectus*	*p* = 0.395 R = −0.228	*p* = 0.636 R = −0.128	*p* = 0.847 R = 0.052	*p* = 0.547 R = −0.163
*Prevotella intermedia*	*p* = 0.413 R = 0.238	*p* = 0.557 R = 0.172	*p* = 0.940 R = 0.022	*p* = 0.420 R = −0.234
*Fusobacterium nucleatum*	*p* = 0.158 R = 0.370	*p* = 0.538 R = 0.166	*p* = 0.111 R = 0.414	*p* = 0.693 R = 0.107
*Prevotella nigrescens*	*p* = 0.625 R = 0.132	*p* = 0.854 R = 0.050	*p* = 0.331 R = −0.260	*p* = 0.134 R = −0.392
*Capnocytophaga ochracea*	*p* = 0.311 R = −0.271	*p* = 0.526 R = −0.171	*p* = 0.243 R = −0.310	*p* = 0.995 R = −0.002
*Capnocytophaga sputigena*	*p* = 0.897 R = 0.035	*p* = 0.373 R = 0.239	*p* = 0.707 R = −0.102	*p* = 0.775 R = −0.078
*Capnocytophaga gingivalis*	*p* = 0.745 R = 0.088	*p* = 0.914 R = 0.029	*p* = 0.098 R = −0.428	*p* = 0.532 R = −0.169

* Spearman’s Correlation Coefficient, ** Correlations are not possible because of constant value of bacterial concentration of 0 CFU/mL.

## Data Availability

The data presented in this study are available from the corresponding author upon request, as they are not publicly available due to privacy or ethical restrictions.
